# Multi-Scale Superpixels Dimension Reduction Hyperspectral Image Classification Algorithm Based on Low Rank Sparse Representation Joint Hierarchical Recursive Filtering

**DOI:** 10.3390/s21113846

**Published:** 2021-06-02

**Authors:** Shenming Qu, Xuan Liu, Shengbin Liang

**Affiliations:** 1 School of Software, Henan University, Kaifeng 475001, China; qsm@vip.henu.edu.cn (S.Q.); xuanliu2612@163.com (X.L.); 2 Institute of Intelligence Networks System, Henan University, Kaifeng 475001, China

**Keywords:** hyperspectral image, low rank sparse representation, entropy rate superpixel segmentation, principal component analysis, domain transform recursive filtering, decision fusion, support vector machine

## Abstract

The original Hyperspectral image (HSI) has different degrees of Hughes phenomenon and mixed noise, leading to the decline of classification accuracy. To make full use of the spatial-spectral joint information of HSI and improve the classification accuracy, a novel dual feature extraction framework joint transform domain-spatial domain filtering based on multi-scale-superpixel-dimensionality reduction (LRS-HRFMSuperPCA) is proposed. Our framework uses the low-rank structure and sparse representation of HSI to repair the unobserved part of the original HSI caused by noise and then denoises it through a block-matching 3D algorithm. Next, the dimension of the reconstructed HSI is reduced by principal component analysis (PCA), and the dimensions of the reduced images are segmented by multi-scale entropy rate superpixels. All the principal component images with superpixels are projected into the reconstructed HSI in parallel. Secondly, PCA is once again used to reduce the dimension of all HSIs with superpixels in scale with hyperpixels. Moreover, hierarchical domain transform recursive filtering is utilized to obtain the feature images; ultimately, the decision fusion strategy based on a support vector machine (SVM) is used for classification. According to the Overall Accuracy (OA), Average Accuracy (AA) and Kappa coefficient on the three datasets (Indian Pines, University of Pavia and Salinas), the experimental results have shown that our proposed method outperforms other state-of-the-art methods. The conclusion is that LRS-HRFMSuperPCA can denoise and reconstruct the original HSI and then extract the space-spectrum joint information fully.

## 1. Introduction

HSI uses numerous continuous narrow-band electromagnetic wave bands to image the surface species and obtain rich joint information of the space-spectrum. With the rapid development of HSI processing and analysis in recent years, HSI classification technology is extensively utilized in agriculture [1], environmental detection [2], marine monitoring [3], and other fields; however, considering the effects of imaging sensor breakdown [4], environmental pollution [5], and other factors, the obtained HSI has mixed noise [6,7,8,9] that reduces the classification accuracy. For example, Tu et al. [7] proposed a kernel entropy component analysis (KECA)-based method for noisy label detection that can remove noisy labels for the PaviaU with 50 true samples and 10 noisy labels per class, KECA obtains 81.11% OA, with 50 true samples and 30 noisy labels per class, the OA is 71%. Xu et al. [8] proposed a spectral-spatial classification of HSI based on low-rank decomposition, by removing the sparse part, the LRD-NWFE-GC [8] obtains 92.3% on Indian Pines even if the training sample is small. On the contrary, the NWFE-GC [8] does not remove the sparse noise, and the classification accuracy is reduced to 74.53%. So, the HSI denoising needs to be solved.

The common noises in HSI are gaussian, impulse, and stripe noises [10]. The methods of noise removal are divided into transform domain filtering and spatial domain filtering [11]. Original spatial domain filtering is easy to perform; however, it will cause a blur of the image and losing some edge and texture details. At present, the edge-preserving filtering method can eliminate the image noise and keep the boundary between various species clear. Typical edge-preserving filtering methods based on spatial domain include a guided filter presented by He et al. [12] and a domain transform recursive filter proposed by Gastal et al. [13]. The non-local mean method is also a type of spatial domain filtering. The related non-local mean algorithms are the shape adaptive non-local mean algorithm [14] and the block matching 3D (BM3D) algorithm [15]. BM3D can preserve the image edge and texture information and obtain a high signal-to-noise ratio [15]. The transform domain filtering denoising transforms the image from the spatial domain to the transform domain through a set of orthogonal transforms. It separates the signal and noise via the different features of noise and signal in the transform domain. In recent years, the denoising algorithm based on sparse representation is extensively used in the field of HSI processing. Low-rank sparse representation is a technique to decompose the observation matrix into the low-rank matrix and sparse matrix, using the low-rank attribute of HSI. This technology can restore the low-rank component robustly when the image is destroyed by mixed noise. In this regard, Zhang et al. [16] proposed an HSI restoration method based on low-rank matrix restoration. A fast denoising algorithm based on low-rank sparse representation was proposed by Lina et al. [17], taking full advantage of the HSIs’ low-rank structure and self-similarity to further improve the denoising effect.

HSI can present rich spectral information by increasing the dimension of HSI data. The classification accuracy will first increase and then decrease, leading to the dimension disaster [18]. The dimension reduction algorithms of HSI are divided into band selection [19,20] and feature extraction. A feature extraction algorithm is divided into supervised and unsupervised. Among the traditional unsupervised algorithms is PCA [21]. PCA does not need label information, it can be reduced to any dimension and is easy to implement. Later, some efficient unsupervised algorithms were proposed, such as subspace feature learning [22] and potential subclass learning [23]. A supervised dimensionality reduction algorithm utilizes supervised information known as labels, to learn the feature space after dimensionality reduction. The representative work includes linear discriminant analysis (LDA) [24] and local Fisher discriminant analysis (LFDA) [25]. LDA cannot only reduce the dimension, but also distinguish different kinds of samples. On the other hand, many HSIs contain complex types of surface features. LDA needs a lot of manpower and costs much time to acquire prior knowledge, and when the ratio between the number of training samples and the number of features is very small, the LDA will achieve lower classification accuracy [24]. In recent years, the spectral features of each pixel are used in most feature extraction algorithms ignoring the spatial features, in order to make full use of spatial features. Kang et al. [26] proposed a spatial-spectral joint classification algorithm based on image fusion (IFRF). An HSI classification algorithm was proposed by Tu et al. [27] combining the correlation coefficient and the joint sparse representation (CCJSR). It can make full use of spectral similarity information and spatial context information at the same time. With the development of deep learning technology, a convolutional neural network (CNN) is extensively utilized in image processing [28]. The relevant CNN classification algorithms based on the spatial-spectral combination include 2D-CNN [29], SSRN [30], CDL-MLR [31] and SSFC [32] to obtain a robust result. Although deep learning methods have great advantages in classification, the architecture of CNNs often has feature redundancy. Therefore, Arijitet et al. [33] proposed a CNN structure based on the GhoMR module to reduce the number of parameters and form a lightweight feature extraction module. Compared with the original 2D-CNN algorithm, the OA of the GhoMR algorithm is improved by 18%, on Indian Pines with 10% training set. Besides, different species often exist in various regions of HSI, resulting in different spectral features. However, most of the extraction algorithms often construct a unified projection space for the feature image after dimensionality reduction [21,26,33], making it impossible to make full use of the different spectral features of various species. To solve this problem, segmentation technology divides the observed image into several specific regions. Jakub et al. [34]. proposed an end-to-end approach to segment hyperspectral images in a fully unsupervised way. Jiang et al. [35]. proposed a feature extraction algorithm based on multi-scale superpixel PCA(MSuperPCA). A superpixel representation based on K-Nearest Neighbor was proposed by Tu et al. [36] for HSI classification (KNNRS). The authors of [34,35,36] fully extracted the spatial-spectral features of HSI.

**Figure 1:**
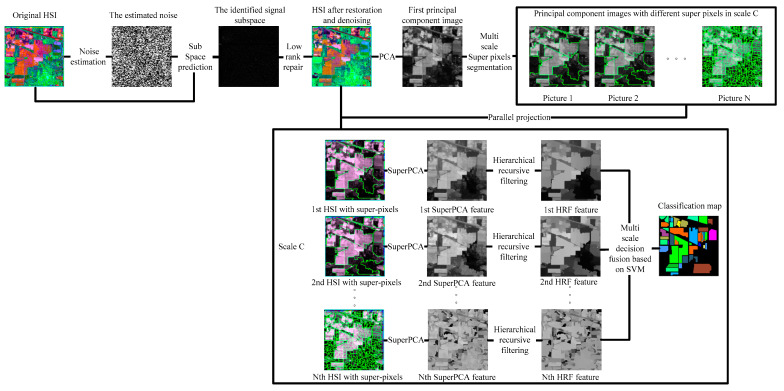
The schematic of the proposed LRS-HRFMSuperPCA method.

Compared with the basic feature extraction algorithm PCA, in recent years, there are many advanced feature extraction algorithms, for example, LFDA [25] and 2D-CNN [29] improve the OA only by at least 3%, IFRF [26], CCJSR [27], SSRN [30], CDL-MLR [31], GhoMR [33], MSuperPCA [35], and KNNRS [36] improve the OA by at least 15% on the Indian Pines with 10% training sets; however, only the spatial domain denoising algorithm is selected for feature extraction [26,36]. The noise in HSI is often mixed noise. Different noise has various imaging reasons and denoising strategies. To reduce the mixed noise of HSI and further enhance the subsequent classification accuracy, transformation domain filtering algorithms based on low-rank sparse representations can robustly recover the low-rank component when the images are destroyed by mixed noise. Nevertheless, HSI classification algorithms only selecting low-rank sparse representations cannot acquire sufficiently spatial structural information, leading to decreased classification accuracy on datasets that are large and complex in texture and structure [27]. The original HSI is not denoised by the HSI feature extraction algorithm based on multi-scale superpixel PCA. Hence, the classification accuracy improvement is not obvious when the training set is large [35]. The problem of feature redundancy of traditional convolutional neural networks is solved by convolutional neural network structure based on the GhoMR module achieving high classification accuracy. However, GhoMR performs PCA on the original HSI and constructs a single projection space, making it impossible to make full use of the spectral features of different species [33]. We propose a multi-scale superpixel unsupervised dimension reduction HSI classification algorithm based on hierarchical recursive filtering and low-rank sparse representation (LRS-HRFMSuperPCA), the schematic diagram is shown in Figure 1. Firstly, the low-rank sparse representation algorithm is used to predict the subspace of HSI. The fully observed HSI is obtained according to the subspace. Next, BM3D is utilized to denoise HSI, and PCA is used to reduce the dimension of HSI after restoration and denoising. The principal component images are segmented by multi-scale entropy rate superpixels. To construct multiple projection spaces, the segmented principal component images with superpixels are projected parallel to the denoised HSIs. The dimension of HSI with superpixels is reduced by PCA again, and each feature image in the multi-scale, after dimension reduction, is transformed by hierarchical recursive filtering. Ultimately, the decision fusion algorithm based on an SVM is used to classify the multi-scale feature images after hierarchical recursive filtering and obtain the final classification result.

The main contributions of LRS-HRFMSuperPCA can be summarized as follows:

1. Compared to other HSI classification algorithms, we propose to integrate the new joint feature extraction framework of spatial domain filtering and transform domain filtering(LRS-HRF) into the MSuperPCA. Firstly, the sparse feature and low-rank structure of HSI are utilized to realize transform domain filtering to separate the mixed noise in the image and improve the PSNR of HSI. Then, the separated feature images are transformed and filtered recursively in a multi-level domain based on the spatial domain. To enhance the spatial-spectral features of the feature image, the strong edges of different objects in the image are preserved and the fine texture structure is removed.
2. The HSI with restoration and denoising is obtained by filtering in the transform domain segmented by multi-scale superpixels, and the low-dimension characteristics of different species in different regions are fully obtained. Then, PCA is performed for each HSI with superpixels in multi-scale, and the spatial information of each HSI is fully utilized.
3. Compared to the state-of-the-art HSI classification algorithms, the LRS-HRFMSuperPCA algorithm achieves the highest OA, AA, and Kappa coefficient on the three real datasets.

## 2. Methodology

### 2.1. Estimation of Noise in Hyperspectral Image

Suppose an original HSI $Y^{L\times Q}\equiv \left[y_{1},y_{2},\ldots ,y_{Q}\right]\in R^{L}$, Q is the number of pixels in each band, L is the number of bands. The high correlation between neighboring spectral bands and the spatial correlation within that band are the reasons underlying the good performance of the linear regression theory in hyperspectral applications [37,38].

Calculate the autocorrelation matrix $\hat{R}:=\left(YY^{T}\right)$ and the inverse matrix $R^{\prime }:={\hat{R}}^{-1}$. Traversing all bands, we assume that $z_{i}$ is explained by a linear combination of the remaining L−1 bands, $z_{i}$ contains the data read by the hyperspectral sensor at the ith band for all image pixels, $Z=Y^{T}$, so we have [39]:

(1) $$z_{i}=Z_{\partial_{i}}\beta_{i}+\xi_{i}$$

where $Z_{\partial_{i}}=\left[z_{1},\ldots ,z_{i-1},z_{i+1},\ldots ,z_{L}\right]$ denotes data from other band image pixels after removing the i-th band, $\beta_{i}$ is the regression vector of size (L−1)×1, and $\xi_{i}$ is the noise vector of size N×1, the least squares estimator of the regression vector $\beta_{i}$ is given by [39]:

(2) $${\hat{\beta }}_{i}={\left(Z_{\partial_{i}}^{T}Z_{\partial_{i}}\right)}^{-1}Z_{\partial_{i}}^{T}z_{i}$$

In order to reduce the computational complexity, define the pseudoinverse $Z_{\partial_{i}}^{\#}={\left(Z_{\partial_{i}}^{T}Z_{\partial_{i}}\right)}^{-1}Z_{\partial_{i}}^{T}$, of size (L−1)×(L−1). Let L×L be the size of symmetric and positive definite matrices R and $R^{-1}$ that are partitioned into block matrices as follows:

(3) $$R=\left[\begin{array}{cc} A & b\\ b^{T} & c \end{array}\right]\;R^{-1}=\left[\begin{array}{cc} A^{\prime } & b^{\prime }\\ {b^{\prime }}^{T} & c^{\prime } \end{array}\right]$$

(4) $${\hat{\beta }}_{i}=Z_{\partial_{i}}^{\#}z_{i}$$

where A and $A^{\prime }$ are (L−1×L−1) matrices, b and $b^{\prime }$ are (L−1×1) vectors, and c and $c^{\prime }$ are scalars. Because they are symmetric and positive, we find:

(5) $$A^{-1}=A^{\prime }-b^{\prime }{b^{\prime }}^{T}/c^{\prime }$$

So, let $A={\left[\hat{R}\right]}_{\partial_{i},\partial_{i}},\;A^{\prime }={\left[R^{\prime }\right]}_{\partial_{i},\partial_{i}},{\left[R^{\prime }\right]}_{\partial_{i},\partial_{i}}$ is the inverse of the autocorrelation matrix obtained by deleting row i and column i, and we have ${\left[\hat{R}\right]}_{\partial_{i},\partial_{i}}{}^{-1}={\left[R^{\prime }\right]}_{\partial_{i},\partial_{i}}-{\left[R^{\prime }\right]}_{\partial_{i},i}{\left[R^{\prime }\right]}_{i,\partial_{i}}/{\left[R^{\prime }\right]}_{i,i}$, the following is the assignment operation, $Z_{\partial_{i}}^{\#}:={\left[\hat{R}\right]}_{\partial_{i},\partial_{i}}{}^{-1}$, $z_{i}:={\left[\hat{R}\right]}_{\partial_{i},i},\;\mathrm{and}\;\mathrm{Equation}\;\left(4\right)$, thus:

(6) $${\hat{\beta }}_{i}:=\left({\left[R^{\prime }\right]}_{\partial_{i},\partial_{i}}-{\left[R^{\prime }\right]}_{\partial_{i},i}{\left[R^{\prime }\right]}_{i,\partial_{i}}/{\left[R^{\prime }\right]}_{i,i}\right){\left[\hat{R}\right]}_{\partial_{i},i}$$

$\hat{\xi_{i}}:=z_{i}-Z_{\partial_{i}}\hat{\beta }$ is calculated for the regression vector $\hat{\beta }$ of each band, the end traversal output $\hat{\xi }:=\left[{\hat{\xi }}_{1},{\hat{\xi }}_{2},\ldots ,{\hat{\xi }}_{L}\right]$ yields the estimated noise in the HSI.

### 2.2. Subspace Prediction of the Signal from the Hyperspectral Image

This section performs a subspace prediction of the signal from the HSI. First, calculate the autocorrelation matrix ${\hat{R}}_{y}\equiv \frac{\left(YY^{T}\right)}{Q}$ and the autocorrelation matrix ${\hat{R}}_{n}:=\frac{\sum_{i}\left({\hat{\xi }}_{i},{\hat{\xi }}_{i}^{T}\right)}{Q}$ of the noise according to the noise vector ${\hat{\xi }}_{i}$. Then, the autocorrelation matrix ${\hat{R}}_{x}:=\frac{\sum_{i}\left(\left(y_{i}-{\hat{\xi }}_{i}\right){\left(y_{i}-{\hat{\xi }}_{i}\right)}^{T}\right)}{Q}$ is computed for the predicted signals. The eigenvectors $E:=\left[e_{1},\ldots ,e_{L}\right]$ for ${\hat{R}}_{x}$ are computed. Given a permutation $\pi =\left\{i_{1},\ldots ,i_{L}\right\}$ of indices i=1,…,L, let us decompose the space ${ℛ}^{L}$ into two orthogonal subspaces. The two orthogonal subspaces are: the k-D subspace $\langle E_{k}\rangle \equiv \left[e_{i_{1}},\ldots ,e_{i_{k}}\right]$ and $\langle E_{\frac{1}{k}}\rangle \equiv \left[e_{i_{k+1}},\ldots ,e_{i_{L}}\right]$, $\langle E_{\frac{1}{k}}\rangle$ is the orthogonal complement of the subspace $\langle E_{k}\rangle$. Let *U_k_* be the projection matrix onto $\langle E_{k}\rangle$ and ${\hat{x}}_{k}\equiv U_{k}y$ be the projection of the observed spectral vector y onto the subspace $\langle E_{k}\rangle$, we introduce the concept of minimum mean square error between x and ${\hat{x}}_{k}$ as a criterion of subspace [40].

(7) $$mse\left(k\right)=tr\left(U_{\frac{1}{k}}R_{y}\right)+2tr\left(U_{k}{\hat{R}}_{n}\right)+c$$

where c is an irrelevant constant, with respect to all the permutations $\pi =\left\{i_{1},\ldots ,i_{L}\right\}$ of size L and to k. So, we can construct the setting item K as follows:

(8) $$mse\left(k\right)=tr\left(U_{\frac{1}{k}}R_{y}\right)+2tr\left(U_{k}{\hat{R}}_{n}\right)+c$$

Let $\hat{k}$ be the number of negative values of $\delta =tr\left(U_{\frac{1}{k}}{\hat{R}}_{y}\right)+2tr\left(U_{k}{\hat{R}}_{y}\right)$, where $\delta :=\left[\delta_{1},\ldots ,\delta_{L}\right]$. Ultimately, the proper subset of feature vector $E:=\left[e_{1},\ldots ,e_{L}\right]$ corresponding to negative $\delta_{i}$ is found from $\delta :=\left[\delta_{1},\ldots ,\delta_{L}\right]$ as the prediction. The value $\hat{S}=\langle [e_{{\hat{i}}_{1}},\ldots ,e_{{\hat{i}}_{\hat{k}}}]\rangle$ of the HSI subspace is retrieved from the signal subspace from $\hat{k}$ and π.

### 2.3. Domain Transform Recursive Filtering (DTRF)

The principle of DTRF is to find a transform $t:R^{2}\to R$ for the input signal I, in the new domain R. It is stated that the Euclidean distance between neighboring samples in the new domain R must equal the $l_{1}$ distance between them in the original domain $R^{2}$ [13].

(9) $$\left|t\left(x_{i},I\left(x_{i}\right)\right)-\left(x_{j},I\left(x_{i}\right)\right)\right|=∥\left(x_{i},I\left(x_{i}\right)\right)-\left(x_{j},I\left(x_{i}\right)\right)∥\;x_{i},x_{j}\subset S\;$$

where $S=\left\{x_{0},x_{1},\ldots ,x_{n}\right\}$ represents the sample set on the signal. Set up ct(x)=t(x,I(x)), h is the sampling interval, and convert Equation (8) to:

(10) ct(x+h)−ct(x)=h+|I(x+h)−I(x)|

In general, to avoid calculating the absolute value, ct is defined as a simple increasing function set up ct(0)=0. We have:

(11) $$ct\left(u\right)=\int_{0}^{u}1+\left|I^{\prime }\left(x\right)\right|dx,u\in \Omega$$

In the actual filtering process, two adjustable parameters are introduced to adjust the filter, the spatial standard deviation $\sigma_{s}$ and the spectral standard deviation $\sigma_{r}$, as:

(12) $$ct\left(u\right)=\int_{0}^{u}1+\frac{\sigma_{s}}{\sigma_{r}}\left|I^{\prime }\left(x\right)\right|dx,u\in \Omega$$

where u is the transform domain signal, $I^{\prime }\left(x\right)$ represents the derivative of input one-dimensional signal I(x). Then, the input signal I is processed by recursive filtering as:

(13) $$K_{i}=\left(1-a^{b}\right)I_{i}+a^{b}K_{j-1}$$

where $a=\exp\left(\frac{-\sqrt{2}}{\delta_{s}}\right)\epsilon \;\left[0,1\right]$ refers to the feedback coefficient, b is the distance between two adjacent samples in the transform domain, $K_{i}$ represents the filtered result. By increasing b, $a^{b}$ tends to be zero, which stops the propagation chain to preserve sharp edges in the signal.

## 3. Proposed LRS-HRFMSuperPCA Method

The HSIs are inevitably contaminated with various noises due to equipment limitation and atmospheric environment impact. On the other hand, the computational complexity will grow exponentially as the dimension increases, which will cause the “curse of dimensionality”; two problems that limit the subsequent classification. We proposed a novel transformation domain-space domain feature extraction framework, called LRS-HRFMSuperPCA, which can effectively improve the classification accuracy and is effective for HSI denoising.

The framework of this joint feature extraction algorithm includes four parts: low-rank sparse representation denoising, multi-scale superpixel dimensionality reduction, and hierarchical domain transform recursive filtering, SVM classification. We detail all parts and provide a pseudo code in the following.

### 3.1. Low-Rank Sparse Representation Denoising Based on MSuperPCA

Transform domain filtering denoising algorithm is used to remove the mixed noise in the original HSI. Following Jiang et al. [35] and Lina et al. [17], we propose the low-rank sparse representation denoising algorithm to remove noise from the original HSI, as the input of the MSuperPCA algorithm. According to Section 2.2, we can obtain the subspace $\hat{S}$ of the original HSI $Y^{L\times Q}$

(14) $$x:=\mathrm{vec}\left(X\right)=\mathrm{vec}\left(\hat{S}Z\right)=\left(I⊗\hat{S}\right)z$$

where ⊗ represents Kronecker product and z:=vec(Z), X is the clean image without noise and I is the identity matrix, Z represents the correlation coefficient matrix. The denoising and inpainting problem is formulated as

(15) $$\underset{z}{\min}\frac{1}{2}∥M\left(I⊗\hat{S}\right)z-y{∥}^{2}+\lambda \phi \left(z\right)$$

where ϕ(z) is the regularization function, λ is the regularization coefficient, y:=vec(Y). Let the mask $M_{i\;}$ acting on the unobserved pixel i and HSI $y_{o,i}$ affected by noise, because $x_{i}=\hat{S}z_{i}$, we have

(16) $$y_{o,i}=M_{i}\hat{S}z_{i}+n_{o,i}$$

In addition, $n_{o,i}$ is the noise part, according to the literature [40], we can obtain ${\hat{z}}_{i}={\left({\hat{S}}^{T}M_{i}^{T}M_{i}\hat{S}\right)}^{-1}{\hat{S}}^{T}M_{i}^{T}y_{o,i}$. By calculating ${\hat{y}}_{i}=\hat{S}{\hat{z}}_{i}$, we can obtain the fully observed HSI $\hat{Y}=\left[{\hat{y}}_{1},{\hat{y}}_{2},\ldots ,{\hat{y}}_{L}\right]$. After recovering the unobserved component, we should solve the denoising problem

(17) $$\underset{Z}{\min}\frac{1}{2}∥\hat{S}Z-\hat{Y}{∥}_{F}^{2}+\lambda \phi \left(Z\right)$$

For the optimization problem of Equation (17), we choose BM3D for denoising [15], because the image obtained by the BM3D algorithm keeps a high PSNR. Then, the correlation coefficient matrix Z is obtained. The HSI $\hat{X}$ is solved by $\hat{X}=\hat{S}Z$.

The dimension of HSI $\hat{X}$ is reduced by PCA, the first principal component images $I_{f}$ are segmented into N principal component images with various superpixel blocks utilizing the entropy rate superpixel algorithm [41], where N=2C+1, C is a user-defined number of scales. The number of superpixels per image in the scale is $S_{c}={\left(\sqrt{2}\right)}^{c}S_{f}$, where c=[−C,−C+1,…,0,…,C−1,C], c denotes the rank of each picture with superpixels, the segmented superpixel blocks of each scale are parallel projected onto the repaired and denoised HSI $\hat{X}$. SuperPCA was performed on $\hat{X}$ [35], therefore, we have

(18) $$F_{i}=SuperPCA\left(J_{m},\hat{X},num\_PC\right)$$

where num _ PC is the dimension after PCA, $J_{m}$ represents the principal component image after superpixel segmentation.

### 3.2. Hierarchical Domain Transform Recursive Filtering (HDTRF)

**Figure 2:**
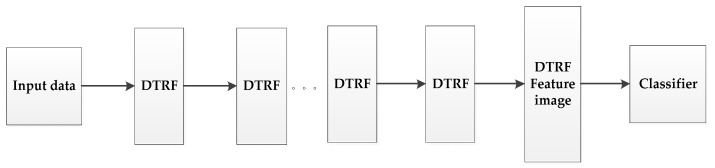
The schematic of the proposed HDTRF method.

Inspired by the idea of deep learning [42,43], we set up a hierarchical structure based on DTRF [13], as shown in Figure 2, the output feature set of the upper level DTRF unit is used as the input of the next level DTRF unit. The spatial information and spectral information of hyperspectral data are fully mined. The obtained spatial-spectral features preserve the global similarity structure, local geometry structure and rich spatial information of the hyperspectral dataset.

### 3.3. Decision Fusion Strategy Based on Support Vector Machine

We use multi-scale entropy rate superpixel segmentation to obtain multiple principal component images. We classify the feature image sets after hierarchical recursive filtering by using SVM. SVM is a supervised learning model, and it is mainly oriented to the linear separable case.

**Figure 3:**
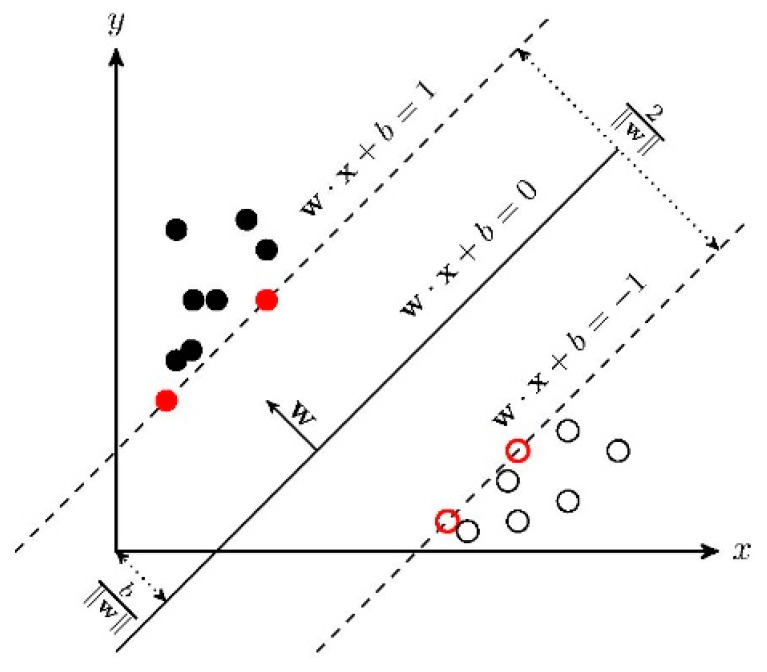
Linear SVM.

In the case of linear inseparability, the linear inseparable samples in the low-dimensional input space are transformed into a high-dimensional feature space by using a nonlinear transformation algorithm. The nonlinear transformation is to map the sample information in the input space into a high-dimensional space through a properly defined inner product function, and then find the optimal linear classification surface in the new space. The optimal classification surface is obtained when the maximum interval classification line is expanded to the n-dimensional space. The optimal classification line cannot only correctly separate some individuals of different categories, but also maximize the overall classification interval. The general principle of SVM is shown in Figure 3.

In the face of the feature classification in HSIs, the problem is always nonlinear. Therefore, we should use some nonlinear feature transformation to map the sample information in the original input space to a high-dimensional feature space, and then find the optimal classification surface in the new space. The objective function is:

(19) $$\min\varphi \left(\omega ,\tau \right)=\frac{1}{2}∥\omega {∥}^{2}+c_{1}\sum_{i=1}^{l}\tau_{i}\;s.t.\;y_{i}\left[w\cdot x_{i}+b\right]-1+\tau_{i}\geq 0,\;\tau_{i}\geq 0\;,i=1,2,\ldots ,n$$

where φ(ω,τ) represents the spatial variation function, $\tau_{i}$ represents the relaxation factor, $c_{1}$ represents the regularization parameter. Facing different classification tasks, we need to construct different kernel functions. The RBF kernel function has the characteristic of unique best approximation. As a kernel function, RBF can map input samples to high-dimensional feature space in SVM. We use the RBF kernel function in the LIBSVM toolbox.

As a feature image can obtain one classification accuracy, we can get 2C+1 classification accuracies from different feature images. The decision fusion algorithm is used to improve classification accuracy. We leverage the majority voting-based decision fusion strategy due to its insensitivity to inaccurate estimates of posterior probabilities, the majority vote is given by [44]:

(20) $$v=arg\underset{i\in \left\{1,2\ldots C\right\}}{max}N\left(i\right),\;\mathrm{where}\;N\left(i\right)=\sum_{j=1}^{2C+1}\alpha_{j}I_{1}\left(v_{j}=i\right)$$

where $I_{1}$ is the indicator function, v is the class label from one of the $C_{1}$ possible classes for the test pixel, j is the classifier index, 2C+1 is the number of classifiers, N(i) is the number of times class i was detected in the bank of classifiers, and $\alpha_{j}$ denotes the voting strength of the j-th classifier. In our method, we use the equal voting strength, $\alpha_{j}=\frac{1}{2C+1}\left(j=1,2,\ldots ,2C+1\right)$.

The detailed procedure for the LRS-HRFMSuperPCA classification is described in Algorithm 1.
**Algorithm 1** LRS-HRFMSuperPCA**Inputs:** Insert original HSI $Y^{L\times Q}$, Y_min is the minimum spectral value of all bands traversed;Step 1 Noise prediction of HSI(1) $Y_{N}=Y^{L\times Q}-Y\_min/\left(Y\_max-Y\_min\right),\;Y_{N}\equiv \left[y_{1},y_{2},\ldots ,y_{Q}\right]$, $Y_{N}\in \left[0,1\right]$;(2) $\hat{R}:=\left(Y_{N}Y_{N}^{T}\right)$,$\;R^{\prime }:={\hat{R}}^{-1}$;(3) for i:=1 to L do(4) ${\hat{\beta }}_{i}:=\left({\left[R^{\prime }\right]}_{\partial_{i},\partial_{i}}-{\left[R^{\prime }\right]}_{\partial_{i},i}{\left[R^{\prime }\right]}_{i,\partial_{i}}/{\left[R^{\prime }\right]}_{i,i}\right){\left[\hat{R}\right]}_{\partial_{i},i}$; $\partial_{i}=1,\ldots ,i-1,i+1,\ldots ,L$
(5) ${\hat{\xi }}_{i}:=z_{i}-Z_{\partial_{i}}{\hat{\beta }}_{i}$
(6) end for(7) Output $\hat{\xi }:=\left[{\hat{\xi }}_{1},{\hat{\xi }}_{2},\ldots ,{\hat{\xi }}_{L}\right]$;Step 2 Subspace prediction of HSI(1) ${\hat{R}}_{y}\equiv \frac{\left(Y_{N}Y_{N}^{T}\right)}{Q}$, ${\hat{R}}_{n}:=\frac{\sum_{i}\left({\hat{\xi }}_{i},{\hat{\xi }}_{i}^{T}\right)}{Q}$, ${\hat{R}}_{x}:=\frac{\sum_{i}\left(\left(y_{i}-{\hat{\xi }}_{i}\right){\left(y_{i}-{\hat{\xi }}_{i}\right)}^{T}\right)}{Q}$;(2) Calculate the eigenvector of ${\hat{R}}_{x}$, $E:=\left[e_{1},\ldots ,e_{L}\right]$;(3) Construction setting item K ,$\;K=\left(\hat{k},\hat{\pi }\right)=arg\underset{k,\pi }{\;min}\left\{tr\left(U_{\frac{1}{k}}{\hat{R}}_{y}\right)+2tr\left(U_{k}{\hat{R}}_{y}\right)\right\}\;$, $U_{k}=E_{k}E_{k}^{T}$;(4) Set $\delta =tr\left(U_{\frac{1}{k}}{\hat{R}}_{y}\right)+2tr\left(U_{k}{\hat{R}}_{n}\right)$*,*
$\delta :=\left[\delta_{1},\ldots ,\delta_{L}\right]$*;*
$\left(\hat{\delta },\hat{\pi }\right):=sort\left(\delta \right)$ by ascending order, save the permutation $\hat{\pi }$; (5) $\hat{k}:=$ number of terms ${\hat{\delta }}_{i}<0\;$, find subspace prediction $\hat{S}=\langle [e_{{\hat{i}}_{1}},\ldots ,e_{{\hat{i}}_{\hat{k}}}]\rangle$Step 3 HSI restoration and denoising(1) Let the mask $M_{i}$ acting on the unobserved pixel i and HSI $y_{o,i}$ affected by noise;(2) The correlation coefficient of the subspace ${\hat{z}}_{i}$ is calculated by ${\hat{z}}_{i}={\left({\hat{S}}^{T}M_{i}^{T}M_{i}\hat{S}\right)}^{-1}{\hat{S}}^{T}M_{i}^{T}y_{o,i}$;(3) Determine the fully observed HSI $\hat{Y}=\left[{\hat{y}}_{1},{\hat{y}}_{2},\ldots ,{\hat{y}}_{L}\right],{\hat{y}}_{i}=\hat{S}{\hat{z}}_{i}$;(4) Solve the optimal value problem of $\underset{Z}{\min}\frac{1}{2}∥\hat{S}Z-\hat{Y}{∥}_{F}^{2}+\lambda \phi \left(Z\right)$ based on the BM3D algorithm; the denoised HSI $\hat{X}$ is obtained by $\hat{X}=\hat{S}Z$;Step 4 Dimension reduction algorithm based on multi-scale superpixels segmentation(1) Insert scale number C *,*
$N=2C+1,C\epsilon N^{*}$; Input initial number of pixels S;(2) Let p=1*,*
$P=pca\left(\hat{X},p\right)$*;* [height,width]=size(P)*;*$\;S_{c}={\left(\sqrt{2}\right)}^{c}S,c=0,\pm 1,\pm 2,\ldots ,\pm C$
(3) for m:=1 to 2C+1 do(4) $J_{m}=seg2bmap\left(mex\_ers\left(P,{\left(\sqrt{2}\right)}^{c}S\right),width,\;height\right)$;(5) end for(6) for k:=1 to N do(7) $F_{k}=SuperPCA(J_{k},\hat{X},num\_PC)$
(8) end for(9) Output $F_{k}=\left[F_{1},F_{2},\ldots ,F_{N}\right]$
Step 5 Decision fusion classification algorithm based on hierarchical recursive filtering(1) for l :==1 to N do(2) $DenoRS_{l}=HRF\left(F_{l},\sigma_{s},\sigma_{r},M\right)$
(3) end for (4) $AC_{l}=SVM\left(DenoRS_{l}\right)$
(5) $v=arg\underset{i\in \left\{1,2\ldots C\right\}}{\;max}N\left(i\right),\;\mathrm{where}\;N\left(i\right)=\sum_{j=1}^{2C+1}\alpha_{j}I_{1}\left(v_{j}=i\right)$


## 4. Experiment and Analysis

### 4.1. Experimental Datasets

**Figure 4:**
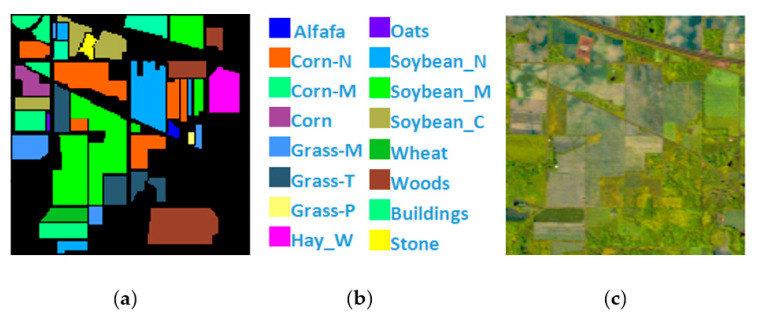
(**a**) Indian Pines ground truth; (**b**) Indian Pines land-cover category; (**c**) Indian Pines false-color image.

Indian Pines is a 145×145 pixel HSI collected by the AVIRIS hyperspectral spectrometer sensor at the Indiana agricultural test site. It contains 220 bands of 16 types of ground objects, and 20 bands with noise removed. The remaining 200 bands are utilized as the experimental dataset, and the total number of labeled pixels is 10,249, Figure 4 shows Indian Pines’ ground true value map, specific species and false color map.

**Figure 5:**
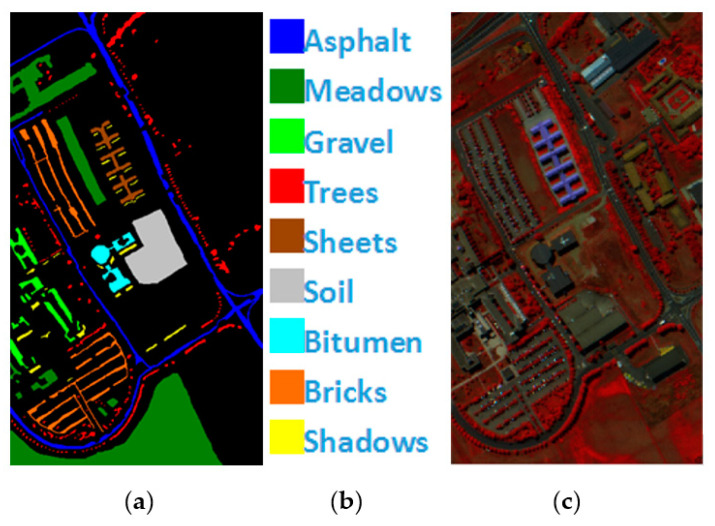
(**a**) PaviaU ground truth; (**b**) PaviaU land-cover category; (**c**) PaviaU false-color image.

PaviaU is a 610×340 pixel HSI collected by the ROSIS hyperspectral spectrometer sensor over Pavia University. A total of 115 bands of 9 types of ground objects are included, and 12 bands with noise are eliminated. The remaining 103 bands are utilized as the experimental dataset, and the total number of labeled pixels is 42,776, Figure 5 shows PaviaU’s ground value map, specific species and false color map.

**Figure 6:**
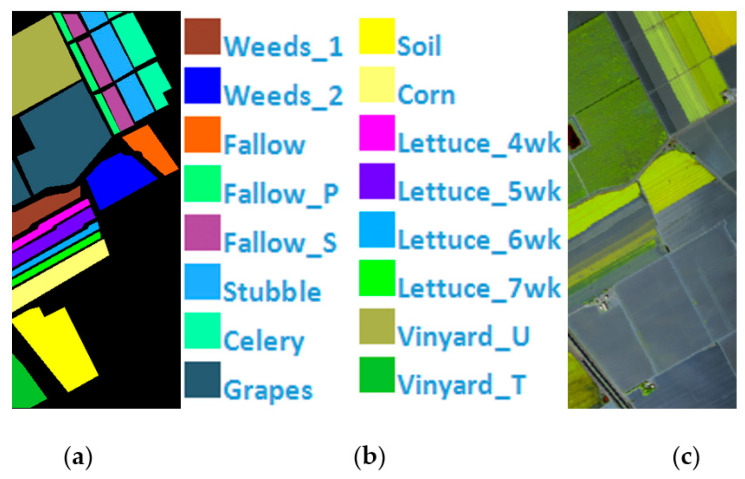
(**a**) Salinas ground truth; (**b**) Salinas land-cover category; (**c**) Salinas false-color image.

Salinas Scene is a 512×217 pixel HSI collected by the AVIRIS hyperspectral spectrometer sensor over the Salinas Valley, California. A total of 224 bands of 16 types of ground objects are included, and 20 bands with noise are eliminated. The remaining 204 bands are employed as the experimental dataset, Figure 6 shows Salinas’ ground value map, specific species and false color map. The total number of labeled pixels is 54,129, Table 1 shows Number of samples in the Indian Pines, PaviaU, and Salinas.

### 4.2. Parameter Analysis

The commonly used HSI classification performance indicators are as follows: OA, AA and Kappa coefficient. Taking the hyperspectral dataset with $C_{1}$ kinds of surface features as an example, by comparing the classification results with the real surface feature distribution data, the confusion matrix $M_{1}\in {\mathbb{R}}^{C_{1}\times C_{1}}$ is obtained, in the matrix, $m_{ij}$ represents the number of samples that belong to class i and are divided into class j. Therefore, the diagonal element $m_{ii}$ in the confusion matrix represents the number of correctly classified samples in each feature category. Next, each evaluation index can be calculated separately.

OA: It refers to the proportion of all correctly classified samples in the total number of samples in the hyperspectral dataset. The calculation formula is:

(21) $$\mathrm{OA}=\frac{\sum_{i=1}^{C_{1}}m_{ii}}{\sum_{i=1}^{C_{1}}\sum_{i=1}^{C_{1}}m_{ij}}$$

AA: According to the OA, the AA is:

(22) $$\mathrm{AA}=\frac{\sum_{i=1}^{C_{1}}\frac{m_{ii}}{\sum_{j=1}^{C_{1}}m_{ij}}}{C}$$

Kappa coefficient: In order to make full use of the information of the confusion matrix to measure the consistency between the classification results and the real surface features distribution map, researchers put forward the concept of Kappa, which is defined as follows:

(23) $$\mathrm{Kappa}=\frac{N_{1}\left(\sum_{i=1}^{C_{1}}m_{ii}\right)-\sum_{i=1}^{C_{1}}\left(\sum_{j=1}^{C_{1}}m_{ij}\sum_{j=1}^{C_{1}}m_{ji}\right)}{N_{1}^{2}-\sum_{i=1}^{C_{1}}\left(\sum_{j=1}^{C_{1}}m_{ij}\sum_{j=1}^{C_{1}}m_{ji}\right)}$$

where, $N_{1}=\sum_{i=1}^{C_{1}}\sum_{j=1}^{C_{1}}m_{ij}$. It represents the total number of samples in the hyperspectral dataset. There are three types of parameters in our paper.

#### 4.2.1. Scale Number C and Initial Superpixel Number S

**Figure 7:**
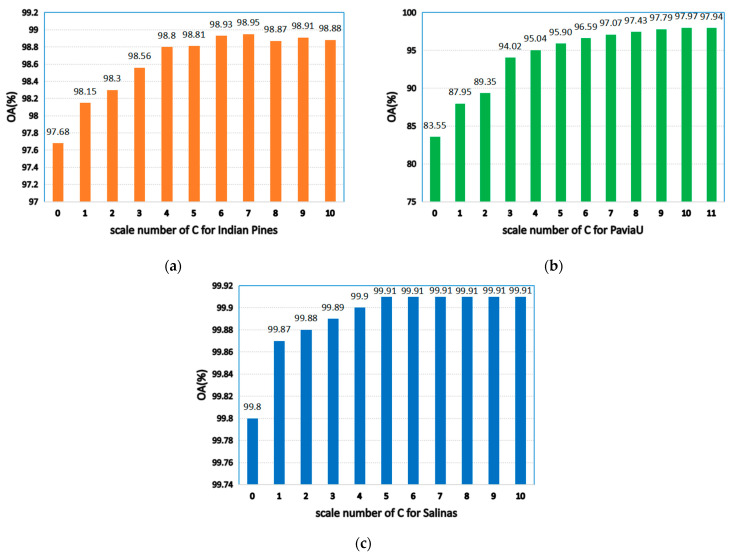
The effects of different scales on classification accuracy of HSIs; (**a**) Indian Pines; (**b**) PaviaU; (**c**) Salinas.

As seen in Figure 7, when C is 0, the model degenerates to a single-scale superpixel dimension reduction. As a result, it is unable to make full use of the spatial information of multiple images, leading to the decline of classification accuracy. When the C value is too large, an over-segmentation will occur. Hence, a single-pixel block is obtained not being visible and the correct label cannot be identified. As shown in Figure 7a, when C is 7, OA will be maximized on the Indian Pines. Figure 7b reveals that, when C is 10, the maximum value is obtained on PaviaU, since the datasets with complex structures and textures and large sizes need to be segmented into more hyper pixels (for instance, PaviaU). Fewer hyper pixels are segmented when the dataset size is smaller (for example, the Indian Pines). 

**Figure 8:**
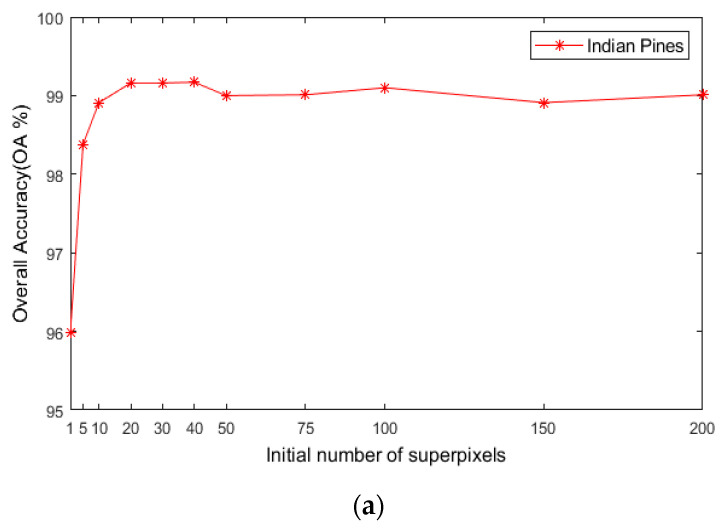

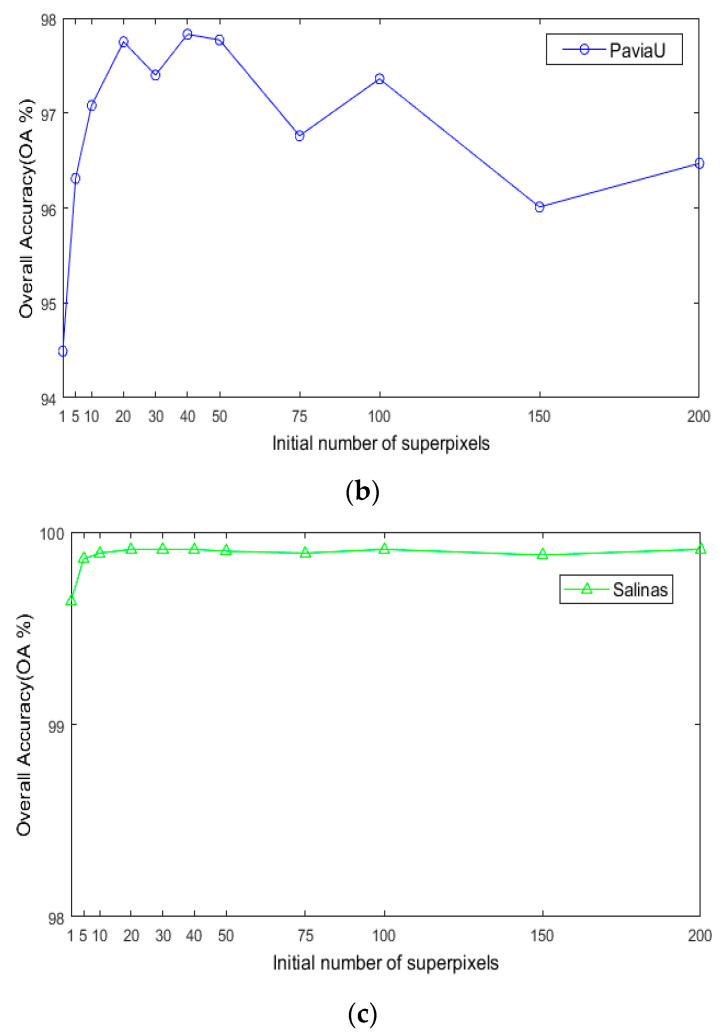
The effects of superpixels on classification accuracy of HSIs; (**a**) Indian Pines; (**b**) PaviaU; (**c**) Salinas.

For the number of initial superpixels S, according to Figure 8, the trend of classification accuracy for the three datasets increases first and then decreases with increasing number of initial hyperpixels. Too few or too many initial superpixels will lead to poor classification accuracy. The reason is that when the initial number of superpixel blocks is too small, the number of superpixel blocks calculated for each image in the scale is also small, and the pixel blocks contain various ground objects, which cannot make full use of the spectral characteristics of different species. When the number of superpixels is too large, a single-pixel block will be smaller, and a single-pixel block will contain fewer pixels and will not be able to utilize all samples belonging to a uniform area. As seen in Figure 8, when S is 40, the maximum OA can be obtained for the three datasets. This indicates that hyperpixel segmentation of the reduced-dimension image can capture the spatial structure of the HSI and improve the OA of the subsequent process.

#### 4.2.2. The Spatial and Range Standard Deviations of the Filter

**Figure 9:**
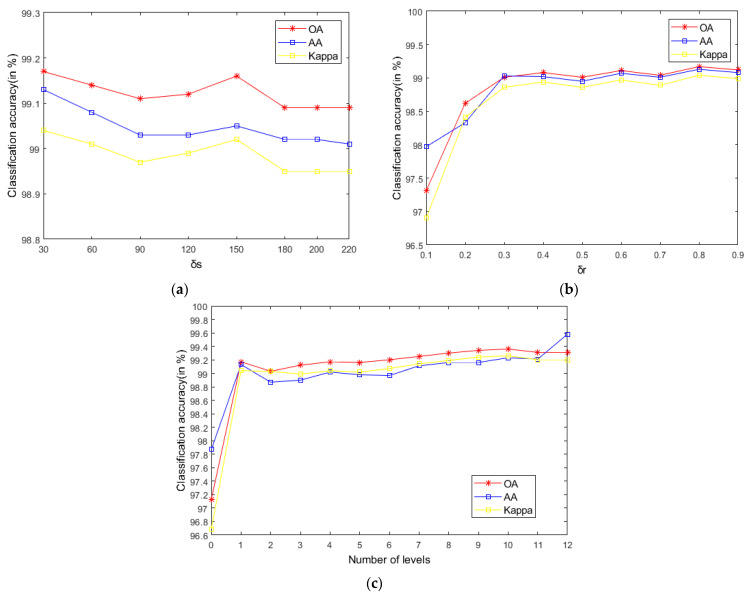
The influence of $\sigma_{s}$, $\sigma_{r}$ and L on the OA of Indian Pines. (**a**)${\;\sigma }_{s}$; (**b**)${\;\sigma }_{r}$; (**c**) L.

**Figure 10:**
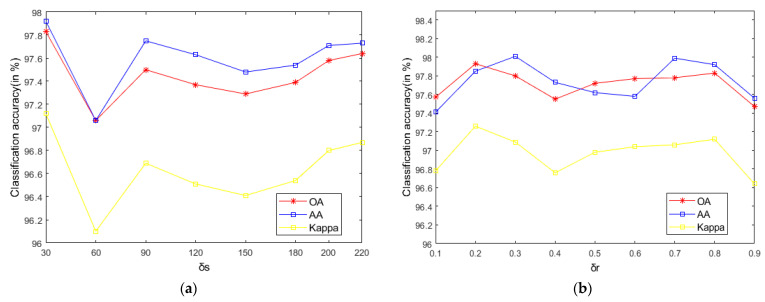

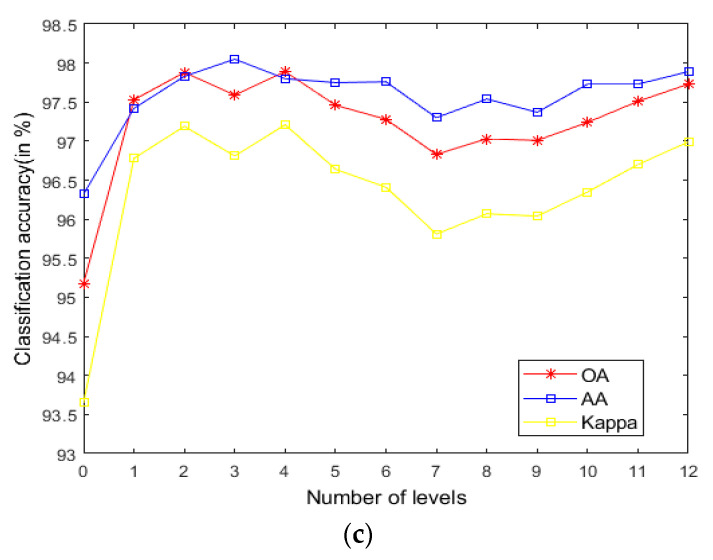
The influence of $\sigma_{s}$, $\sigma_{r}$ and L on the OA of PaviaU. (**a**)${\;\sigma }_{s}$; (**b**)${\;\sigma }_{r}$; (**c**) L.

**Figure 11:**
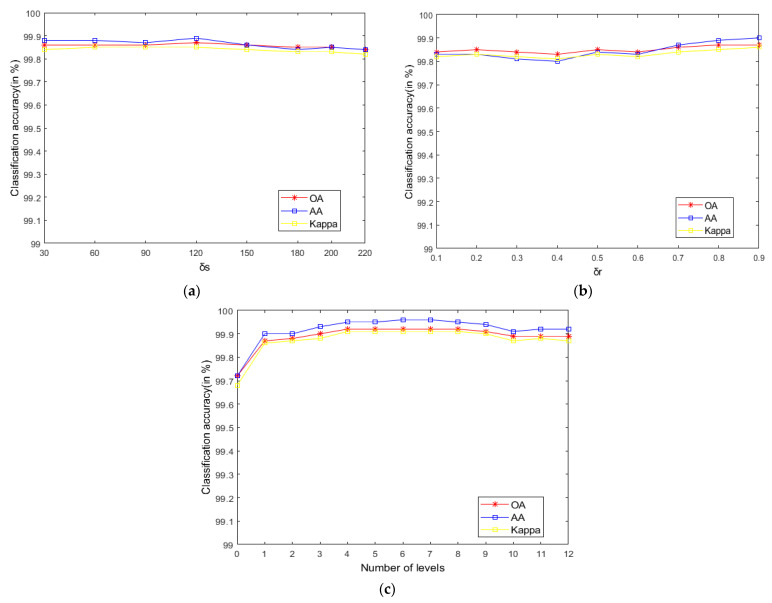
The influence of $\sigma_{s},{\;\sigma }_{r}$ and L on the OA of Salinas. (**a**)${\;\sigma }_{s}$; (**b**)${\;\sigma }_{r}$; (**c**) L.

As seen in Figure 9, on the Indian Pines, when $\sigma_{s}$ is 30 and $\sigma_{r}$ is 0.8, the method can obtain the optimal classification accuracy. This will not be repeated for the Salinas and PaviaU. For the recursive layer number L, when the recursion level is 0, the LRS-HRFMSuperPCA method degenerates to the LRS-MSuperPCA method leading to the failure to guarantee similar eigenvalues for the adjacent pixels on the same side of the edge and the classification accuracy is minimum, when the recursion level is 1, the fine texture structure cannot be completely eliminated by single-layer recursive filtering. Hence, it eliminates the fine texture structure and preserves the edges. This can be inferred from the SuperPCA part in schematic Figure 1. According to Figure 9, Figure 10 and Figure 11, it is indicated that the proposed HDTRF can enhance the classification accuracy of HSI.

#### 4.2.3. Kernel Function Parameter G and Regularization Parameter $c_{1}$ in SVM

**Figure 12:**
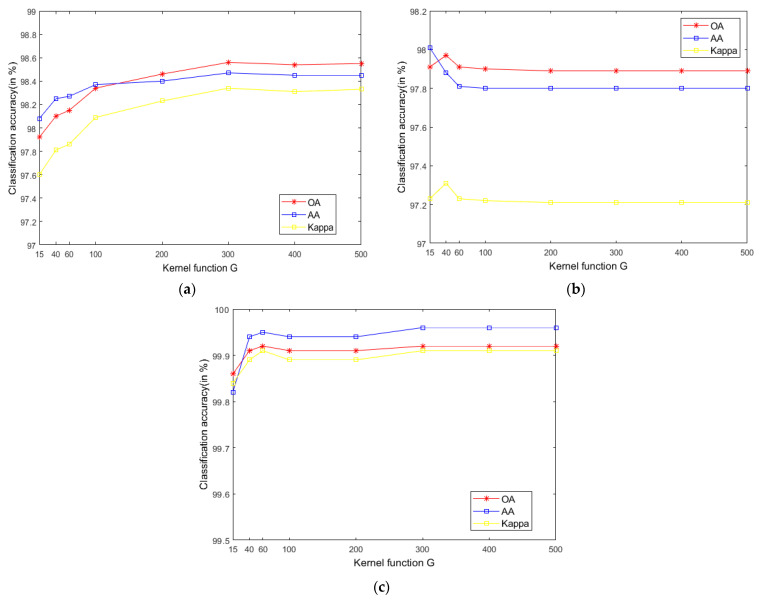
The effect of the different kernel function G in SVM on classification accuracy; (**a**) Indian Pines; (**b**) PaviaU; (**c**) Salinas.

**Figure 13:**
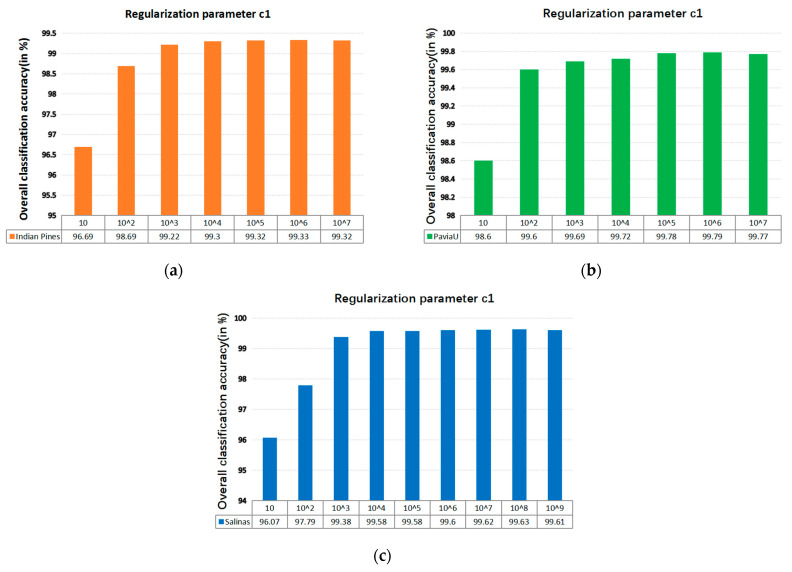
The effect of the different regularization parameter $c_{1}$ in SVM on OA; (**a**) Indian Pines; (**b**) PaviaU; (**c**) Salinas.

The SVM classifier used in the LRS-HRFMSuperPCA technique utilizes the LIBSVM toolbox in MATLAB. Moreover, the comparison algorithm of the SVM classifier also uses the LIBSVM toolbox in MATLAB containing the kernel parameter gamma G and regularization parameter $c_{1}$. Figure 12 and Figure 13 represent the effect of different kernel parameters G and regularization parameter $c_{1}$ on classification accuracy in three datasets, respectively. It is observed that for the three datasets, the maximum classification accuracy can be achieved when the kernel parameter G is 300, 40 and 400, respectively, and the different regularization parameter $c_{1}$ is set to 10^6^, 10^6^ and 10^8^. In our classifier, a higher gamma value G leads to a higher dimension of the mapping, and the training result is better. We choose the higher $c_{1}$, as the higher $c_{1}$ aims to classify all training samples correctly by giving the model the freedom to choose more samples as support vectors. Since the Salinas training sample is larger than the Indian Pines and PaviaU, we can see that the value of $c_{1}$ will increase with the increase of training set samples.

The experimental parameter settings of the LRS-HRFMSuperPCA method in subsequent experiments are shown in Table 2.

### 4.3. Experimental Results and Analysis

**Figure 14:**
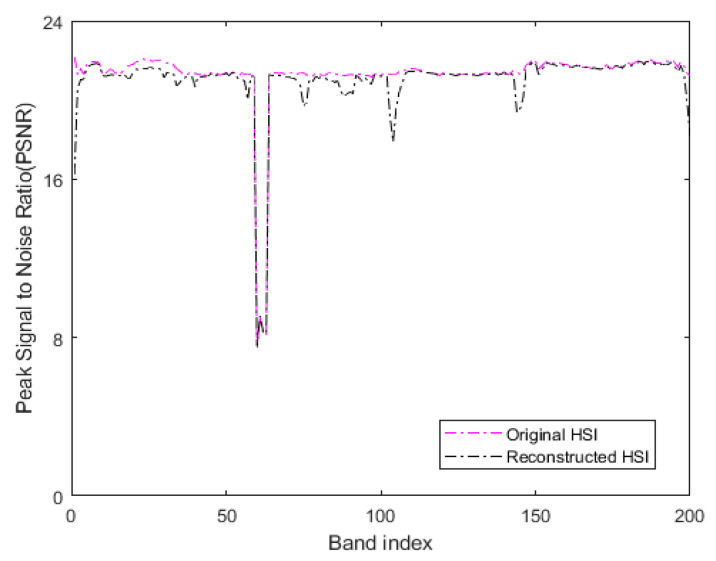
The PSNR values of all bands of original HSI and denoised reconstructed HSI on the Indian Pines.

The HSI restored by the transform domain filtering algorithm based on low-rank sparse representation is better than the original image. In our paper, the peak-signal-to-noise ratio (PSNR) was utilized to compare the quality of the denoised HSI and the original HSI, as follows:

(24) $$\mathrm{PSNR}=20\cdot log_{10}^{\left(\frac{MAX_{I}}{\sqrt{MSE}}\right)}$$

(25) $$MSE=\frac{1}{mn}\sum_{i=0}^{m-1}\sum_{j=0}^{n-1}{\left[I\left(i,j\right)-K\left(i,j\right)\right]}^{2}$$

where $MAX_{I}$ is the maximum value of the color of the image point, MSE represents the mean square error between two monochromatic images I and K of *m∗n* size. In our method, the original HSI with additive Gaussian noise with a noise variance $\sigma^{2}$ of 0.01 is used as the reference image to determine the PSNR. In the experiment, the original HSI and the denoised HSI obtained by low-rank sparse representation are considered as the processed images. Figure 14 represents the PSNR values of all bands of the original HSI and the HSI denoised by low-rank sparse representation on the Indian Pines. Table 3 denotes the average PSNR values of all bands of the two HSIs after summation.

When comparing the PSNR values, the reference image used in our method is a noisy HSI with additive Gaussian noise. Thus, the greater the MSE between the processed image and the reference image, the greater the difference between the processed image and the reference image. Moreover, the smaller the corresponding PSNR value, in our method, the smaller the difference between the processed image and the reference image damaged by additive noise. The larger corresponding PSNR value indicates that the quality of the processed image is poor. As seen in Figure 14, the PSNR values of the reconstructed HSI obtained by our method are lower compared to the original HSI in all bands. The third row of Table 3 reveals that the OA of the LRS-HRFMSuperPCA is better than that of the HRFMSuperPCA without low-rank sparse representation denoising on the 10% training set. Therefore, the transform domain filtering denoising algorithm based on low-rank sparse representation of HSI can enhance the quality of the original HSI and improve the OA.

The LRS-HRFMSuperPCA is compared with several HSI classification models, including original classification algorithm, SVM, PCA and the recently proposed IFRF [26], CCJSR [27], MSuperPCA [35], SSRN [30], KNNRS [36] and GhoMR [33]. To avoid the chance of the experimental results, the average value of five runs is considered as the final result, including our algorithm and all other comparison algorithms. At the same time, for a fair comparison, the parameters of the comparison algorithm are set as the default optimal parameters based on the relevant literature. Table 4 represents the specific experimental parameters of the comparison algorithm. The experimental environment of the LRS-HRFMSuperPCA method and other comparative algorithms is a notebook with 12 GB memory, Intel Core i5 2.2 GHz CPU and the development environment in MATLAB R2018a is used. GhoMR and SSRN are performed utilizing PyTorch 1.6.0 with CUDA 10.1 in the open-source GPU environment of Google Colaboratory. The code for the LRS-HRFMSuperPCA is available at https://gitee.com/lxxds123/my-dem (accessed on 25 April 2021).

In order to fully compare with the training set in the references corresponding to the comparison algorithm, the contrast experiments are carried out on the training set which accounts for $t_{1}$ = 77 (10% Training set) and $t_{1}$ = 173 (20% Training set) for per class on Indian Pines and $t_{2}$ = 475 (10% Training set) and $t_{2}$ = 1139 (20% Training set) for PaviaU, $t_{3}$ = 102 (3% Training set) and $t_{2}$ = 170 (5% Training set) on Salinas, leaving the rest samples to form the testing samples. For 10% training data on Indian Pines and PaviaU, 3% training data on Salinas, from Table 5, our method obtains 99.32%, 99.87% and 99.73% OA values on the three datasets. For 20% training data on Indian Pines and PaviaU, 5% training data on Salinas, from Table 6, our method obtains 99.63%, 99.91% and 99.90% OA values on the three datasets.

To compare 10%, 20%, 3% and 5% of the total randomly selected training samples, the eight advanced comparison algorithms in Table 4 and the LRS-HRFMSuperPCA method are used. In this section, only the comparison results on the Indian Pines are discussed. Table 7 shows that, when the training set is 10%, the OA of our method is improved by 17.65% and 20.27%, respectively, compared with the original SVM and PCA on the Indian Pines. Compared to the IFRF, according to Table 3, the low-rank sparse representation denoising algorithm can enhance the quality of the original HSI, thus improving the OA of 0.9% on 10% of the Indian Pines. Compared to the CCJSR, the OA of our method is enhanced by 3.32% and 2.78% over the two training sets on Indian Pines. In comparison to the MSuperPCA, when the training set is 20%, the OA of our method on the Indian Pines dataset is improved by 1.78%. The reason is that our method combines the spatial domain filtering algorithm and the transform domain filtering algorithm. The mixed noise can be removed by the transform domain filtering denoising algorithm based on low-rank sparse representation within the original HSI. At the same time, the small-scale structure of the HSI can be eliminated by the multi-level spatial domain recursive filtering algorithm fully obtaining the spatial-spectral joint information of the HSI and improving the OA. Compared to KNNRS, the OA of our method is always higher than that of the LRS-HRFMSuperPCA on the three datasets (from Table 7 and Table 8). Compared to GhoMR, the single projection space constructed by the GhoMR makes it impossible to make full use of the spectral features of different species. Our method can randomly control the scale of multi-scale superpixel segmentation, and each obtained superpixel block is a set of pixels with similar characteristics. Subsequently, a hierarchical recursive filtering algorithm is used to extract the spatial-spectral joint information in each pixel block region to improve the consistency and similarity within the region. At the same time, our method runs in a non-GPU environment. Compared to the GhoMR running on GPU, we use CPU to reduce the experimental equipment cost.

**Figure 15:**
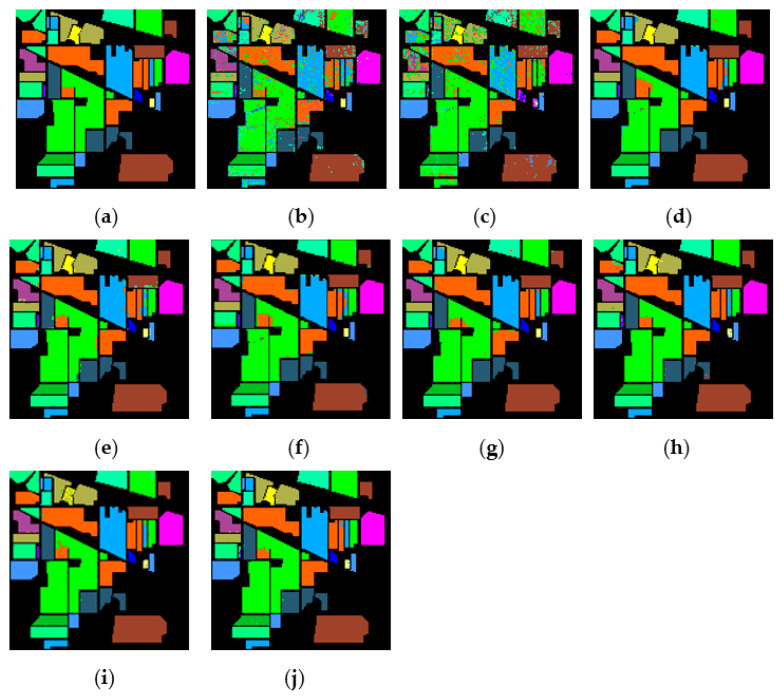
The classification maps obtained by different methods on the Indian Pines when training samples account for 10% of the reference. (**a**) GroundT. (**b**) SVM (OA = 81.99%). (**c**) PCA (OA = 79.58%). (**d**) IFRF (OA = 97.91%). (**e**) CCJSR (OA = 96.87%). (**f**) MSuperPCA (OA = 97.76%). (**g**) SSRN (OA = 98.73%). (**h**) KNNRS (OA = 99.18%). (**i**) GhoMR (OA = 98.66%). (**j**) LRS-HRFMSuperPCA (OA = 99.34%).

**Figure 16:**
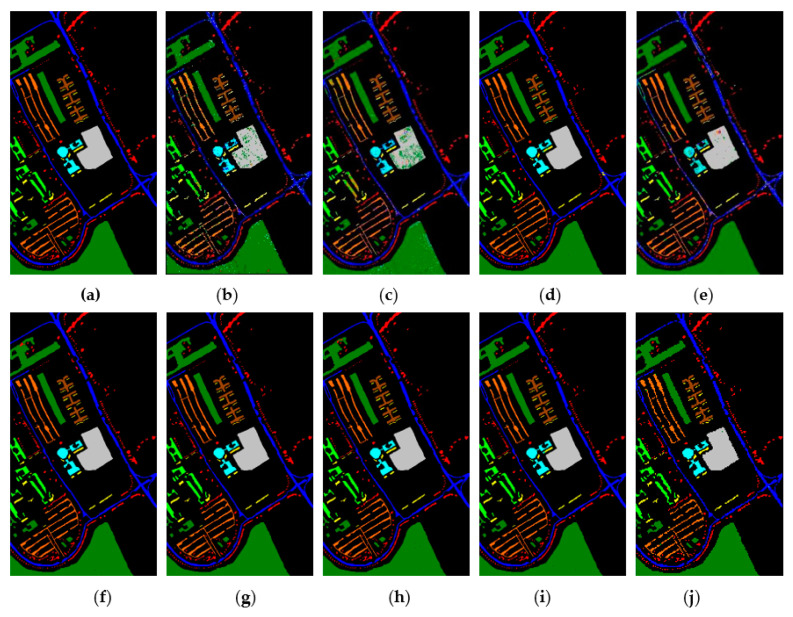
The classification maps obtained by different methods on the PaviaU when training samples account for 10% of the reference. (**a**) GroundT. (**b**) SVM (OA = 94.17%). (**c**) PCA (OA = 90.50%). (**d**) IFRF (OA = 99.21%). (**e**) CCJSR (OA = 93.73%). (**f**) MSuperPCA (OA = 99.40%). (**g**) SSRN (OA = 99.47%). (**h**) KNNRS (OA = 99.45%). (**i**) GhoMR (OA = 99.54%). (**j**) LRS-HRFMSuperPCA (OA = 99.88%).

**Figure 17:**
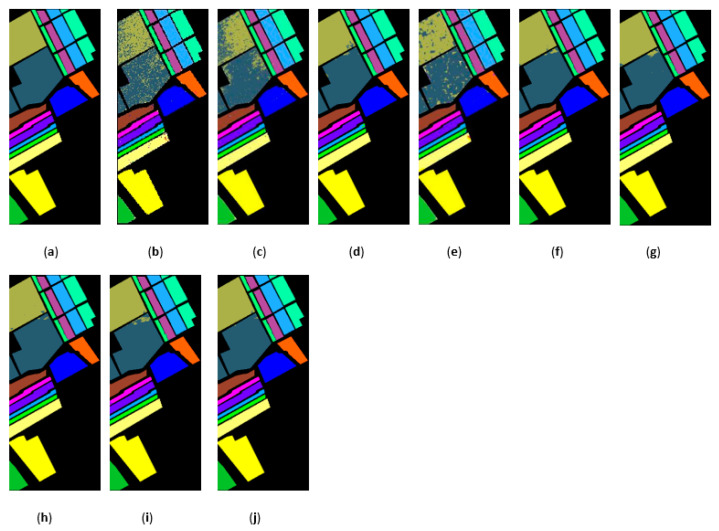
The classification maps obtained by different methods on the Salinas dataset when training samples account for 3% of the reference. (**a**) GroundT. (**b**) SVM (OA = 93.05%). (**c**) PCA (OA = 92.94%). (**d**) IFRF (OA = 99.42%). (**e**) CCJSR (OA = 95.37%). (**f**) MSuperPCA (OA = 99.53%). (**g**) SSRN (OA = 99.34%). (**h**) KNNRS (OA = 99.68%). (**i**) GhoMR (OA = 99.30%). (**j**) LRS-HRFMSuperPCA (OA = 99.78%).

Figure 15, Figure 16 and Figure 17 represent the random feature classification map obtained by our method and different comparison algorithms and the OA of the corresponding feature classification map. As seen in Figure 15, the OA of the SVM and PCA is not ideal since they do not combine the joint information of spatial-spectrum, and the “salt and pepper” noise phenomenon appears in the classification result graph. In contrast, IFRF and KNNRS combined with the spatial-spectrum can effectively eliminate the noise pixels, and the “salt and pepper” noise phenomenon is greatly reduced. Compared to PCA and SVM, the OA of IFRF is improved by 18.33% and 15.92%, respectively, and that of KNNRS is improved by 17.3% and 19.6%, respectively. According to the surface features classification map of Indian Pines, the OA of our method is 1.43%, 2.47%, and 0.16% higher than that of IFRF, CCJSR, and KNNRS. Compared to the advanced MSuperPCA, SSRN and GhoMR algorithms, the OA of our algorithm on the Indian Pines is improved by 1.58%, 0.61% and 0.68%, respectively. Improvements or comparable results are obtained on PaviaU and Salinas as well that are not repeated here.

**Figure 18:**
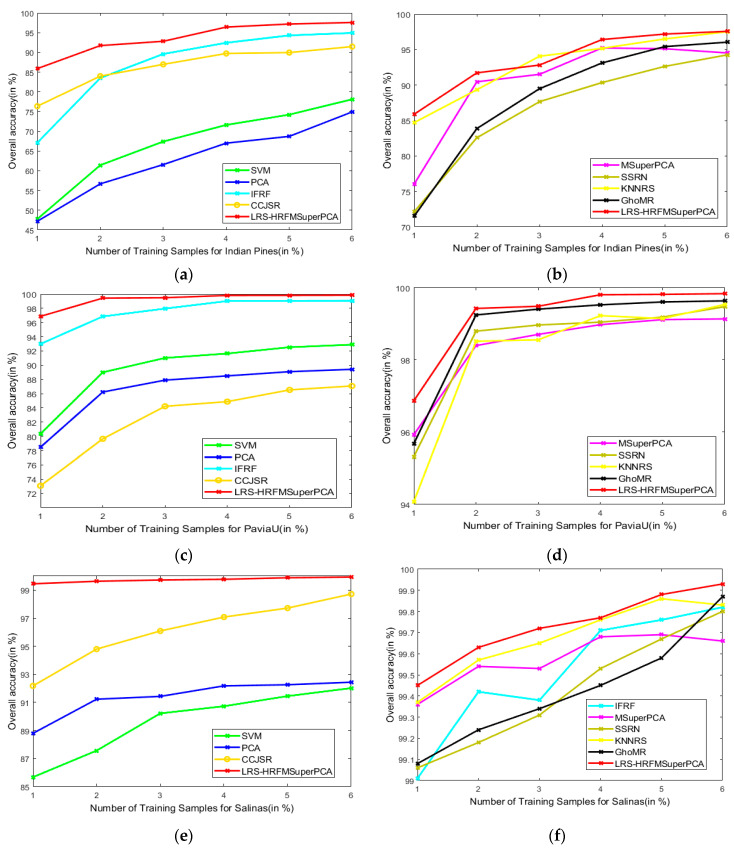
OA of the proposed LRS-HRFMSuperPCA method with different numbers of training samples on different images. (**a**,**b**) Indian Pines; (**c**,**d**) PaviaU; (**e**,**f**) Salinas.

This section analyzes the effects of various training and test sets on the OA of our method and the other methods. Figure 18 represents the classification results of the LRS-HRFMSuperPCA method. The number of training samples of three datasets increased from 1% to 6%. According to Figure 18, with different training sample numbers, the OA can be improved significantly by our method by increasing the number of training samples. Hence, we can conclude that our method can achieve the best OA for the three datasets compared to other state-of-the-art comparison algorithms.

The computational time of the LRS-HRFMSuperPCA algorithm and other comparison algorithms are represented in Table 9. The running environment of the LRS-HRFMSuperPCA and all the comparison algorithms is a notebook computer with 12 GB memory and a 2.2 GHz CPU processor. Except for GhoMR and SSRN, the running environment of GhoMR and SSRN are pytorch 1.6.0 and CUDA10.1 in the open-source GPU environment of Google lab, the running environment of other algorithms is MATLAB2018a. Table 8 shows the number of training samples as a percentage of the total sample number in the measurement run time to experiment on the three datasets. According to the analysis of Table 9, the LRS-HRFMSuperPCA algorithm takes a long time on the Indian Pines. The main reason is that this technique utilizes multi-level domain transform recursive filtering and multi-scale superpixel segmentation. As seen in Figure 18a,b, under different training sets of Indian Pines, the OA of our method is enhanced compared to other comparison algorithms. On the PaviaU and Salinas, the CCJSR took the most time since the computation of the correlation coefficient of the training set increased by increasing the training set, followed by the KNNRS. The main computation of the KNNRS is caused by the operation of KNNs. The operation time of the MSuperPCA is mainly caused by multiscale hyperpixel segmentation of HSIs. The run time of our method on the PaviaU is lower compared to the KNNRS, MSuperPCA, and CCJSR. The OA index of this method is also improved compared to other algorithms on PaviaU, for the GhoMR. Although the time cost of our algorithm on Indian Pines and PaviaU is higher than that of the GhoMR, the classification accuracy of our algorithm is better than that of the GhoMR in various training sets. Moreover, the time cost on Salinas is lower than that of GhoMR. Thus, the experimental results show that the run time of LRS-HRFMSuperPCA is acceptable.

## 5. Discussion

We reveal some interesting points about the LRS-HRFMSuperPCA methods by conducting several comparison experiments and parameter discussion on three HSIs.

**Figure 19:**
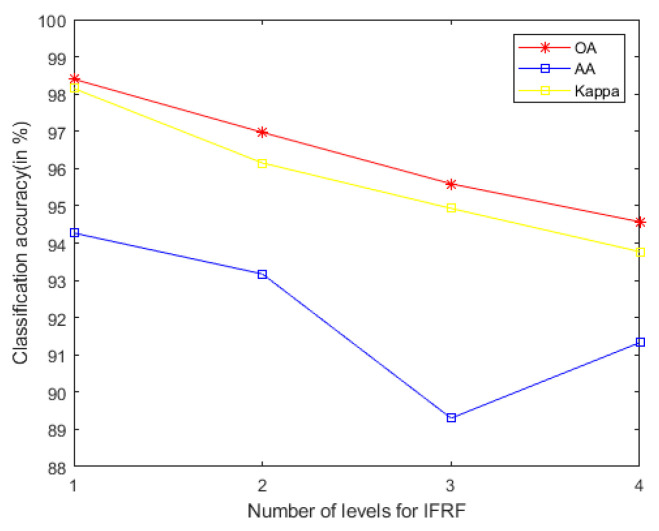
The accuracy of image fusion and recursive filtering algorithm (IF gets non-low-rank feature image) under adding the influence of hierarchical filtering on Indian Pines.

**Figure 20:**
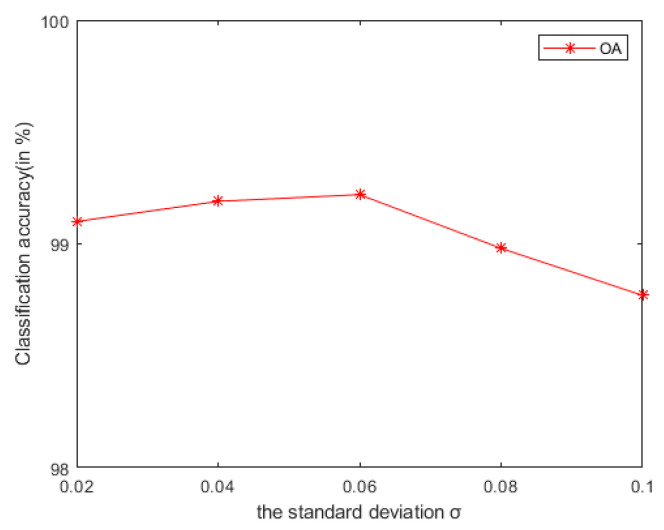
Under different additive Gaussian noise environments, the OA of LRS-HRFMSuperPCA algorithm.

- In general, we notice that the mixed noise in HSI will reduce the classification accuracy [9]. Considering the effects of imaging sensors breakdown, environmental pollution, and other factors, these degradation factors account for a small proportion in HSI. We usually build them as sparse noise models here. A clear natural image (specific to our article, it is a HSI) is often a low-rank structure; therefore, when dealing with the mixed noise problem of natural image, we usually use the low-rank sparse representation algorithm to solve it. In order to remove the small-scale textures in HSI, domain transform recursive filtering is used to solve this problem. Another knowledge base is that after the stable low-rank feature natural image (specific to our article, it is a HSI) is extracted (specific to our article, it is LRS-MSuperPCA in Section 3.1), the joint information of the spatial-spectrum of the feature image can be obtained by hierarchical filtering (specific to our article, it is HDTRF in Section 3.2). In order to facilitate understanding, we apply hierarchical recursive filtering to IFRF (Figure 19) for experimental verification.
  Compared with the LRS-HRFMSuperPCA algorithm, using hierarchical filtering in Figure 9c, with the increase of the level, the OA of the three kinds of classification in Figure 19 decreases significantly. The experimental results show that our knowledge base is correct.
- Based on the two knowledge bases proposed in the previous point, denoising based on low-rank sparse representation (let it set to function A) belongs to the transform domain filtering algorithm, while denoising based on hierarchical recursive filtering (let it set to function B) belongs to the spatial domain filtering algorithm. Let us set the penalty factor ρ that controls the proportion between function A and function B, ρ∈[0,1], the optimal filtering of the finding F can be expressed as a function, which can be used for future research to find an optimal framework:
  (26) F=ρ×A+(1−ρ)B
- According to the data in Table 7 and Table 8, LRS-HRFMSuperPCA is superior to the feature extraction algorithm using a single filter. Compared with IFRF and KNNRS, our method uses low-rank sparse representation algorithm to denoise the original HSI. It can be seen from Table 3 that the OA of LRS-HRFMSuperPCA is 0.23% higher than that of HRFMSuperPCA, and PSNR has also improved. This supports the correctness of LRS [17] that can remove mixed noise in the original HSI. Compared with LRS-MSuperPCA, the small-scale textures of the HSI can be eliminated by the multi-level spatial domain recursive filtering algorithm, fully obtaining the spatial-spectral joint information of the HSI and improving the OA. It can be seen from Figure 9c, Figure 10c and Figure 11c that the OA of LRS-HRFMSuperPCA is always higher than that of LRS-MSuperPCA on the three datasets. The comparative experiments verify that the DTRF [13] and IFRF [26] can remove texture structure and improve classification accuracy. These results support earlier studies.
- Our finding is a novel feature extraction framework with joint low-rank sparse representation-hierarchical domain transform recursive filtering. The framework agrees with the existence of mixed noise in the original HSI [9] and the spectral vectors live in a low-dimensional subspace [45]. The proposed framework is applied to HSI feature extraction (MSuperPCA), so the algorithm LRS-HRFMSuperPCA is obtained. As can be seen from Figure 1, we use hierarchical recursive filtering to extract features images from HSI. According to the experimental results in Table 7 and Table 8, it improves the OA, AA and Kappa. In the field of remote sensing science, there are two expectations for remote sensing image classification [46]: the first is feature mining, and the second is to design a classifier with high classification accuracy. Our LRS-HRFMSuperPCA algorithm achieves the two expectations.
- Compared with the HSI classification algorithm based on non-deep learning [26,27,35,36] and deep learning [30,33], the advantage of the LRS-HRFMSuperPCA algorithm is the use of the dual filtering framework. Specifically, compared with IFRF [26] and KNNRS [36] which only use spatial domain filtering, LRS-HRFMSuperPCA uses spatial-transform dual domain filtering, LRS-HRFMSuperPCA reconstructs the original HSI by recovering the pixels not observed in some known bands. We conduct an experiment to prove the anti- noise performance of the algorithm, in which 0-means additive Gaussian noise is added to the original HSI. According to Figure 20, with the increase of Gaussian noise intensity, our algorithm still maintains an OA of more than 98%, accounting for 10% of the Indian Pines samples on the training sets. Compared with CCJSR [27], which only uses a sparse representation, LRS-HRFMSuperPCA uses hierarchical domain transformation recursive filtering to make full use of spatial context information. Compared with SSRN [30] and GhoMR [33], the two space-spectrum joint classification algorithms based on deep learning use a single projection space. They cannot make full use of the low-dimensional characteristics of each species. Multi-scale superpixel segmentation can learn the low-dimensional features of different regions in HSIs by inputting the number of scales. At the same time, the architectures of SSRN [30] and GhoMR [33] need expensive GPU hardware to train and store. LRS-HRFMSuperPCA uses CPU to reduce the consumption of the experimental equipment. However, we find that most feature extraction algorithms (such as PCA in LRS-HRFMSuperPCA) can compress the information transformation of the original data into the low-dimensional feature space and this may lose the physical meaning of the original feature. In future research, we will suggest to focus on the method of unsupervised band selection algorithm instead of SuperPCA. Band selection selects a desired band subset from the original bands and preserves well the spectral meaning of spectral channels. In practice, prior knowledge about the surface features are difficult to obtain, so it is necessary to develop an unsupervised feature extraction algorithm. Specifically, for the reconstructed HSI with superpixels $\hat{X}$, hybrid scheme-based methods implement multiple schemes to select appropriate bands from $\hat{X}$. We can try to integrate both clustering and ranking based techniques to search for the best combination of bands with higher classification accuracy. Another major drawback of our method is that its performance is still affected by the number of superpixel segmentation and the shape of superpixel in our method is irregular. Specifically, once pixels of different classes exist in the same superpixel region, the pixels in these superpixels cannot be effectively classified. In future research, for different categories of pixels in the same superpixel, we will segment all kinds of pixels again, according to the weight of each pixel in the superpixel. The weight can be measured by relative entropy or Euclidean distance, etc. On the other hand, a gradient-based superpixel method has the advantage of regular shape and controllable number of generated superpixels, which is the direction to solve the problem of irregular shape of superpixels in the future.

## 6. Conclusions

In this paper, a simple and effective HSI classification algorithm is proposed combining transform domain filtering and spatial domain filtering. The objective of our method is to combine the PCA based on multi-scale entropy rate superpixel dimension reduction within two types of joint filtering denoising framework. To remove the mixed noise in the original HSI, the denoising algorithm based on a low-rank sparse representation is used to denoise and restore the original HSI, improve the peak-signal-to-noise ratio of the original HSI, and the subsequent classification accuracy. A multi-scale entropy ratio-based superpixel segmentation algorithm is utilized to segment the repaired image. By dividing the whole HSI into several regions, the same superpixel block has similar reflection characteristics, which is convenient for subsequent dimension reduction processing and finding the low-dimensional features of the HSI. Secondly, hierarchical recursive filtering is applied to the feature images after SuperPCA is making full use of the spatial information contained in HSI. Experiments on three real HSIs reveal that the proposed LRS-HRFMSuperPCA algorithm enhances the classification accuracy compared with the original MSuperPCA algorithm and the recently proposed HSI classification algorithm. However, the disadvantage of the LRS-HRFMSuperPCA algorithm is that the parameters of the algorithm are manually selected, thereby reducing the operability of the algorithm. Therefore, in future research, we will focus on how to automatically determine the optimal parameters of our algorithm.

## Figures and Tables

**Table 1:** Number of samples in the Indian Pines, PaviaU, and Salinas.

Indian Pines		PaviaU		Salinas	
Class Names	Numbers	Class Names	Numbers	Class Names	Numbers
Alfalfa	46	Asphalt	6631	Weeds_1	2009
Corn-N	1428	Meadows	18,649	Weeds_2	3726
Corn-M	830	Gravel	2099	Fallow	1976
Corn	237	Trees	3064	Fallow_P	1394
Grass-M	483	Sheets	1345	Fallow_S	2678
Grass-T	730	Soil	5029	Stubble	3959
Grass-P	28	Bitumen	1330	Celery	3579
Hay_W	478	Bricks	3682	Grapes	11,271
Oats	20	Shadows	947	Soil	6203
Soybean_N	972			Corn	3278
Soybean_M	2455			Lettuce_4wk	1068
Soybean_C	593			Lettuce_5wk	1927
Wheat	205			Lettuce_6wk	916
Woods	1265			Lettuce_7wk	1070
Buildings	386			Vinyard_U	7268
Stone	93			Vinyard_T	1807
Total Number	10,249	Total Number	42,776	Total Number	54,129

**Table 2:** The experimental parameters settings in LRS-HRFMSuperPCA.

Datasets	C	S	$\sigma_{s}$	$\sigma_{r}$	L	G	$c_{1}$
Indian Pines	7	40	30	0.8	10	300	10^6^
PaviaU	10	40	30	0.2	4	40	10^6^
Salinas	5	40	120	0.9	6	400	10^8^

**Table 3:** The average PSNR of the original Indian Pines and the reconstructed Indian Pines in all bands and the OA of the LRS-HRFMSuperPCA method and HRFMSuperPCA method are compared.

	Original Indian Pines	Reconstructed Indian Pines
Average value of PSNR	21.26 dB	20.96 dB
OA(10% Train set)	99.04%(HRFMSuperPCA)	99.27%(LRS-HRFMSuperPCA)

**Table 4:** The application of experimental parameters in the comparison algorithm.

Method	Parameters or Structure Used in the Comparison Algorithm
SVM	Penalty parameter c=100, Kernel function parameter G=0.125.
PCA(SVM)	Feature dimension of Indian Pines after dimension reduction D=100, the D of PaviaU and Salinas are 70 and 120, Penalty parameter c=3000, Kernel function parameter $G=2^{-7}$.
IFRF(SVM)	The parameters of recursive filtering are set to $\delta_{s}$=200 and $\delta_{r}=0.3$. The number of features k is set 20, penalty parameter c=100, kernel function parameter G=4.
CCJSR	$\mathrm{The}\;\mathrm{relationship}\;\mathrm{among}\;\mathrm{OA},\;\mathrm{the}\;\mathrm{joint}\;\mathrm{neighboring}\;\mathrm{scale}\;S_{e}$, $\mathrm{and}\;\mathrm{the}\;\mathrm{sparsity}\;\mathrm{level}\;S_{l}$, the number of nearest neighbors N, and the regularized parameter λ. $\mathrm{When}\;S_{e}$$\mathrm{is}\;\mathrm{set}\;\mathrm{to}\;6\;\mathrm{and}\;S_{l}$is set to 2, λequals 0.6 and N is set to 4. The highest classification accuracy is obtained by the proposed method.
MSuperPCA(SVM)	The scale number of C for Indian Pines, PaviaU, and Salinas is set to 4, 6, and 4, respectively. The number of superpixels is set to the initial optimal value, S = 100, S = 20, and 100 for Indian Pines, PaviaU, and Salinas, for SVM, penalty parameter $c=10^{5}$, Kernel function parameter G=500.
SSRN	The SSRN includes two convolutional layers and two spectral residual blocks as a spectral feature learning section, a 3-D convolutional layer and two spatial residual blocks as a spatial feature learning section, an average pooling layer, and a fully connected layer.
KNNRS	The parameters of recursive filtering are set to $\delta_{s}$=204 and $\delta_{r}=0.6$. The parameters of KNN are set to $k_{1}=1$ and $k_{2}=9$. The number of superpixels N for Indian Pines, PaviaU, and Salinas is set to 3300, 8000, and 9000.
GhoMR	The GhoMR have two hyperparameters—number of Ghost transformations (T) and spatial size of ghost filters ($K_{T}$), for Indian Pines T=2 and $K_{T}=3$, for PaviaU T=4 and $K_{T}=7$, for Salinas T=2 and $K_{T}=3$

**Table 5:** The data distribution along with class-wise accuracies, OA, AA, Kappa on Indian Pines, PaviaU, and Salinas datasets, respectively, for randomly chosen 10% training data on Indian Pines and PaviaU, 3% training data on Salinas, leaving the rest samples to form the test set.

Indian Pines (10% Training Data)				PaviaU (10% Training Data)				Salinas (3% Training Data)			
Name	Training	Test	Accuracy	Name	Training	Test	Accuracy	Name	Training	Test	Accuracy
Alfalfa	23	23	100	Asphalt	475	6156	99.69	Weeds_1	102	1907	100
Corn-N	77	1351	98.67	Meadows	475	18,174	99.92	Weeds_2	102	3624	100
Corn-M	77	753	100	Gravel	475	1624	99.88	Fallow	102	1874	100
Corn	77	160	100	Trees	475	2589	99.61	Fallow_P	102	1292	100
Grass-M	77	406	99.75	Sheets	475	870	100	Fallow_S	102	2576	99.42
Grass-T	77	653	100	Soil	475	4554	99.98	Stubble	102	3857	99.95
Grass-P	14	14	100	Bitumen	475	855	99.77	Celery	102	3477	99.91
Hay_W	77	401	100	Bricks	475	3207	99.91	Grapes	102	11,169	99.91
Oats	10	10	100	Shadows	475	472	100	Soil	102	6101	100
Soybean_N	77	895	100					Corn	102	3178	99.93
Soybean_M	77	2378	98.70					Lettuce_4wk	102	966	100
Soybean_C	77	516	97.87					Lettuce_5wk	102	1825	99.45
Wheat	77	128	99.22					Lettuce_6wk	102	814	99.16
Woods	77	1188	100					Lettuce_7wk	102	968	100
Buildings	77	309	99.68					Vinyard_U	102	7166	99.21
Stone	47	46	100					Vinyard_T	102	1705	99
OA(%)			99.32	OA(%)			99.87	OA(%)			99.73
AA(%)			99.62	AA(%)			99.86	AA(%)			99.75
Kappa			0.9921	Kappa			0.9982	Kappa			0.9970

**Table 6:** The data distribution along with class-wise accuracies, OA, AA, Kappa on Indian Pines, PaviaU, and Salinas datasets, respectively, for randomly chosen 20% training data on Indian Pines and PaviaU, 5% training data on Salinas, leaving the rest samples to form the test set.

Indian Pines (20% Training Data)				PaviaU (20% Training Data)				Salinas (5% Training Data)			
Name	Training	Test	Accuracy (%)	Name	Training	Test	Accuracy (%)	Name	Training	Test	Accuracy (%)
Alfalfa	23	23	100	Asphalt	1139	5492	99.84	Weeds_1	170	1839	100
Corn-N	173	1255	99.60	Meadows	1139	17510	99.94	Weeds_2	170	3556	100
Corn-M	173	657	100	Gravel	1050	1049	99.90	Fallow	170	1806	100
Corn	119	118	100	Trees	1139	1925	99.58	Fallow_P	170	1214	100
Grass-M	173	310	100	Sheets	673	672	100	Fallow_S	170	2508	99.80
Grass-T	173	557	100	Soil	1139	3890	99.97	Stubble	170	3789	99.84
Grass-P	14	14	100	Bitumen	665	665	99.85	Celery	170	3409	99.94
Hay_W	173	305	100	Bricks	1139	2543	99.96	Grapes	170	11101	99.93
Oats	10	10	100	Shadows	474	473	99.79	Soil	170	6033	100
Soybean_N	173	799	100					Corn	170	3108	99.97
Soybean_M	173	2282	99.12					Lettuce_4wk	170	898	100
Soybean_C	173	420	99.29					Lettuce_5wk	170	1757	99.94
Wheat	103	102	99.02					Lettuce_6wk	170	746	99.87
Woods	173	1092	100					Lettuce_7wk	170	900	100
Buildings	173	213	99.53					Vinyard_U	170	7098	99.59
Stone	47	46	100					Vinyard_T	170	1637	100
OA(%)			99.63	OA(%)			99.91	OA(%)			99.90
AA(%)			99.79	AA(%)			99.87	AA(%)			99.93
Kappa			0.9957	Kappa			0.9986	Kappa			0.9988

**Table 7:** OA, AA, and Kappa using the LRS-HRFMSuperPCA and other state-of-the-art methods on 10% and 20% randomly chosen training samples.

Train	Methods	Indian Pines			Percentage of OA Value Increase (%)	PaviaU			Percentage of OA Value Increase (%)
		OA (%)	AA (%)	Kappa		OA (%)	AA (%)	Kappa	
10%	SVM	81.67	79.84	0.7876	17.65	91.08	93.39	0.8791	8.79
	PCA	79.05	77.02	0.7602	20.27	90.74	88.05	0.8763	9.13
	IFRF	98.42	97.80	0.9825	0.90	99.14	98.73	0.9906	0.73
	CCJSR	96.00	95.44	0.9478	3.32	93.66	90.80	0.9160	6.21
	MSuperPCA	96.37	97.96	0.9582	2.95	99.37	99.42	0.9914	0.50
	SSRN	98.65	98.53	0.9817	0.67	99.79	99.66	0.9972	0.08
	KNNRS	99.24	96.99	0.9913	0.08	99.32	99.11	0.9905	0.55
	GhoMR	98.64	98.00	0.9845	0.68	99.75	99.33	0.9967	0.12
	LRS-HRFMSuperPCA	99.32	99.62	0.9921	/	99.87	99.86	0.9982	/
20%	SVM	86.24	83.15	0.8427	13.39	95.20	93.60	0.9363	4.72
	PCA	82.64	80.52	0.7947	16.99	91.21	88.42	0.8829	8.71
	IFRF	98.85	98.62	0.9866	0.78	99.41	99.16	0.9911	0.51
	CCJSR	96.85	96.22	0.9641	2.78	96.28	93.67	0.9508	3.64
	MSuperPCA	97.85	97.96	0.9749	1.78	99.61	99.60	0.9944	0.31
	SSRN	99.45	99.52	0.9937	0.18	99.87	99.81	0.9989	0.05
	KNNRS	99.31	96.62	0.9922	0.32	99.53	99.18	0.9936	0.39
	GhoMR	99.54	99.30	0.9947	0.09	99.90	99.82	0.9986	0.02
	LRS-HRFMSuperPCA	99.63	99.79	0.9957	/	99.92	99.88	0.9988	/

**Table 8:** OA, AA, and Kappa using the LRS-HRFMSuperPCA and other state-of-the-art methods on 3% and 5% randomly chosen training samples on Salinas.

Train	Methods	Salinas			Percentage of OA Value Increase (%)
		OA (%)	AA (%)	Kappa	
3%	SVM	85.96	89.87	0.8429	13.77
	PCA	91.74	95.25	0.9097	7.99
	IFRF	99.28	99.20	0.9916	0.45
	CCJSR	95.74	97.00	0.9554	3.99
	MSuperPCA	99.53	99.37	0.9948	0.20
	SSRN	99.41	99.36	0.9933	0.32
	KNNRS	99.72	99.74	0.9969	0.01
	GhoMR	99.37	99.61	0.9932	0.36
	LRS-HRFMSuperPCA	99.73	99.75	0.9970	/
5%	SVM	87.48	91.36	0.8602	12.42
	PCA	92.36	95.86	0.9148	17.54
	IFRF	99.75	99.72	0.9969	0.15
	CCJSR	97.85	97.86	0.9765	2.05
	MSuperPCA	99.69	99.59	0.9965	0.21
	SSRN	99.60	99.58	0.9953	0.30
	KNNRS	99.79	99.75	0.9977	0.11
	GhoMR	99.77	99.81	0.9974	0.13
	LRS-HRFMSuperPCA	99.90	99.93	0.9988	/

**Table 9:** The computational time (in seconds) of different methods (10% training set).

Dataset (10%)	SVM	PCA	IFRF	CCJSR	MSuperPCA	SSRN	KNNRS	GhoMR	LRS-HRFMSuperPCA
Indian Pines	20	3	18	490	141	74	69	178	347
PaviaU	55	11	117	5977	1661	106	1684	680	1618
Salinas	139	23	82	10582	707	332	4525	860	762

## Data Availability

The data presented in this study are available on request from the corresponding author.
